# Expanding invasive species impact assessments to the ecosystem level with EEICAT

**DOI:** 10.1371/journal.pbio.3003665

**Published:** 2026-03-10

**Authors:** Laís Carneiro, Daniel Pincheira-Donoso, Boris Leroy, Sandro Bertolino, Morelia Camacho-Cervantes, Ross N. Cuthbert, Alok Bang, Jane A. Catford, Josie South, Steven J. Cooke, Elena Angulo, Franck Courchamp

**Affiliations:** 1 Université Paris–Saclay, CNRS, AgroParisTech, Ecologie Société Evolution, Gif-sur-Yvette, France; 2 School of Biological Sciences, Queen’s University Belfast, Belfast, United Kingdom; 3 Biologie des Organismes et des Ecosystèmes Aquatiques, Department Adaptation du Vivant, Museum National d’Histoire Naturelle, Paris, France; 4 Department of Life Sciences and Systems Biology, University of Turin, Torino, Italy; 5 Invasive Species Ecology Lab, Institute of Marine Sciences & Limnology, Universidad Nacional Autonoma de Mexico, Mexico City, Mexico; 6 Institute for Global Food Security, School of Biological Sciences, Queen’s University Belfast, Belfast, United Kingdom; 7 Biology Group, School of Arts and Sciences, Azim Premji University, Bhopal, India; 8 Department of Geography, King’s College London, London, United Kingdom; 9 Fenner School of Environment & Society, The Australian National University, Canberra, ACT, Australia; 10 Centre for Invasion Biology, School of Biology, Faculty of Biological Sciences, University of Leeds, Leeds, United Kingdom; 11 South African Institute for Aquatic Biodiversity (SAIAB), Makhanda, South Africa; 12 Water Leeds, School of Biology, Faculty of Biological Sciences, University of Leeds, Leeds, United Kingdom; 13 Canadian Centre for Evidence-Informed Conservation, Carleton University, Ottawa, Ontario, Canada; 14 Estación Biológica de Doñana, CSIC, Seville, Spain

## Abstract

The ecological impacts of biological invasions vary widely in type, scale, and severity, highlighting the need for consistent assessment tools. The Environmental Impact Classification for Alien Taxa (EICAT) provides a standardized framework for assessing their effects, but focuses mainly on population-level impacts. We introduce the Extended EICAT (EEICAT), which incorporates impacts across three ecological dimensions, from individuals to ecosystems, with an impact-based approach. EEICAT enables classification of 19 impact types at the invasion-event level, making it suitable for primary research, synthesis, and management. This framework aims to improve the detection, comparison, and communication of complex ecological impacts caused by biological invasions.

## Abstract 2

This Consensus View introduces EEICAT, a framework for assessing how biological invasions affect species, ecosystems, and the environment. It covers 19 types of impacts and aims to help scientists and managers compare and address invasion-driven changes.

## Introduction

Invasive alien species are among the main drivers of ecological and socio-economic impacts (Box 1) worldwide [1–4]. The impacts caused by invasive alien species (invasive species hereafter; Box 1) vary widely depending on their traits, the newly established interactions with native species, and the suite of abiotic conditions they encounter in the new habitat [5–7]. These diverse impacts arise from multiple mechanisms (Box 1), such as predation, competition, and disease transmission, that exert both direct and indirect effects on native species and ecosystems. This often results in complex outcomes that can be positive or negative for nature and people [4,8,9].

**Box 1. Glossary**

**Impact**

A measurable change to the properties of an ecosystem caused by an invasive species in any environment [5]. We do not consider socio-economic impacts in this framework, nor do we distinguish between positive and negative effects.

**Invasive species**

An alien or non-native species that is transported beyond its natural biogeographic range. Once established and spread, they are usually referred to as invasive species. Here, we consider that any species can cause impacts regardless of the stage of invasion, and refer to them all as invasive species throughout the text.

**Impact mechanisms**

The processes through which an invasive species exerts its impact. There are at least 12 mechanisms: competition, predation, hybridization, transmission of disease, parasitism, poisoning/toxicity, bio-fouling or other direct physical disturbance, grazing/herbivory/browsing, chemical impact, physical impact, structural impact, and indirect impacts through interactions with other species. The definitions for all mechanisms can be found in the Environmental Impact Classification for Alien Taxa (EICAT) and EICAT+ [10,11].

**Ecological level**

There are six ecological levels: individuals, population, species, assemblage, ecosystem functions, and abiotic components.

**Ecological dimension**

Refers to the three groups of ecological levels: individuals and populations, species and assemblages, and ecosystem function and abiotic environment. We use this term to separate the three columns where impacts can be classified.

**Impact magnitude category**

When a study has enough information to be considered for evaluation, we can classify each ecological impact type into one of the five different levels of impact magnitude, from Minimal to Massive.

**Invasion event**

The occurrence of non-native species in a defined spatial unit (e.g., grid cell, region, island, lake) at a given point in time, whether through introduction, establishment, or spread. Each species × recipient system × context combination constitutes one invasion event.

**Impact event**

Each impact identified from an Invasion event. It is the combination of the invasive species × recipient system × context x impact type. Multiple impact events can be reported for a single invasion event.

Assessing the effects of invasive species is essential for developing effective management and mitigation strategies. However, most of the assessed evidence focuses on impacts in just a few ecological levels (Box 1) — mostly populations and, to some extent, assemblages — whereas assessments of ecosystem-level impacts remain limited and fragmented [12]. This includes studies of changes in ecosystem functions and the abiotic environment, which are essential for fully understanding the extent to which invasions drive ecological change and the magnitude of their impacts [5,13–15]. Indeed, among the 19 types of impact caused by biological invasions [8], 12 concern assemblages, ecosystem and abiotic levels.

The most widely used framework for assessing the ecological impacts of invasion is the Environmental Impact Classification for Alien Taxa (EICAT) [10,16,17]. EICAT provides a standardized, evidence-based methodology for assessing the nature and severity of environmental impacts caused by invasive species within their introduced range. It has been applied in different contexts, such as national lists [18] or protected areas [19], for different taxonomic groups, such as global assessments of 415 bird species [20] and 352 insect species [21], and more recently in the IPBES Thematic Assessment Report on Invasive Alien Species and their Control [4]. In EICAT, the magnitude of impacts from a given invasive species are attributed after compiling evidence of their effects on native species at the individual or population levels, whereas impacts at higher ecological levels (e.g., assemblages, ecosystems and habitat/abiotic environment) are not sufficiently addressed. Changes in communities can be inferred from the loss of at least one native species, and alterations in physico-chemical characteristics or habitat structure are considered, but they are limited to an impact classification only if they directly affect native species. Invasive species impacts beyond the individual or population levels are, however, numerous and important [5,12]. For example, lake trout (*Salvelinus namaycush*) has disrupted food webs in Yellowstone National Park in the USA, altering both biotic interactions and the abiotic environment [22,23]. The smooth cordgrass (*Spartina alterniflora*) introduced in China’s coastal regions, outcompetes native plants and significantly changes habitat structure and ecosystem functions [24]. This includes impacts on sediment dynamics, nutrient cycling, and hydrological processes while also reducing biodiversity in affected areas.

Emerging evidence also highlights the cross-ecosystem impacts of invasive species. These can modify flows of energy and matter between ecosystems that are often considered separately in management planning, such as the quality, magnitude, and novelty of resource flows between terrestrial and aquatic systems [25–27]. Accounting for these overlooked impacts has significant implications for both research and the development of holistic management strategies. This calls for the adaptation of impact assessment tools to better identify and quantify the magnitude of such effects.

Fortunately, the structure of the current, much-used EICAT framework allows for a straightforward extension to integrate the effects of biological invasions on ecosystem structure and functions. In this Consensus View, we propose a framework adapted from EICAT that includes all impacts on abiotic components and ecosystem functions, services, and structure. Considering the widespread use and adoption of EICAT, we expanded its impact magnitude classification system to capture the currently missing ecological dimensions (Box 1). Building on a recently developed typology of invasion impacts [8], we thus propose the Extended Environmental Impact Classification for Alien Taxa (EEICAT), an improved invasion impact assessment procedure that explicitly incorporates all of the 19 types of impacts of biological invasions.

## EEICAT framework conceptualization and expansion

The EEICAT framework was designed through expert elicitation conducted during two dedicated workshops in 2023 and 2024 with a large and diverse group of experts in biological invasions, including researchers, scholar-practitioners, and managers from around the globe. We aimed to build on the existing EICAT framework by addressing identified current gaps and expanding its applicability. Specifically, we identified and categorized all known types of ecological impacts caused by invasive species [8], grouped these impacts according to their relevance at different ecological dimensions, and evaluated how concepts from the EICAT framework apply to all types of impacts in each dimension, adapting them when necessary.

We introduced two additional dimensions to separate the impacts into three categories: (i) individual and population, (ii) species and assemblage, and (iii) ecosystem function and abiotic. The five original EICAT impact magnitude categories (Box 1), minimal concern (MC), minor (MN), moderate (MO), major (MR), and massive (MV) [10,28], are retained and adapted across each dimension. This results in a matrix of 15 possible impact classifications, enabling the assessment of each type of impact identified for a given biological invasion event (Box 1; Table 1).

**Table 1:** The EEICAT framework.

Impact category	Individual and population	Species and assemblage	Ecosystem function and abiotic
**Minimal concern (MC)**	No detectable impact on individuals or populations	No detectable impact on species range or assemblages	No detectable impact on ecosystem function or abiotic components
**Minor impact (MN)**	Detectable impact on individuals, but not on populations	Detectable impact on species range, but not on assemblages	Detectable impact on abiotic components, but not on ecosystem function
**Moderate impact (MO)**	Large impact on individuals and/or detectable impact on populations	Large impact on species range and/or detectable impact on assemblages	Impact on abiotic components affecting native biota and/or detectable impact on ecosystem function
**Major impact (MR)**	Large but reversible impact on populations	Large but reversible impact on assemblages	Large but reversible impact on ecosystem function and/or abiotic components
**Massive impact (MV)**	Irreversible impact on populations	Irreversible impact on assemblages	Irreversible impact on ecosystem and/or abiotic components

Moreover, our framework allows multiple impacts reported within a single study to be classified independently at each impact level. To achieve this, we use the typology that identifies 19 different impact types across six ecological levels [8]. It is important to note that, unlike EICAT, the mechanisms through which the invasive species affect the native species or environment are not a requirement for this classification. Indeed, EEICAT aims for a broader assessment that primarily considers the ecological impact at the respective ecological dimension and impact magnitude category. This increases the breadth of evidence that can be integrated into impact assessments for any given species invasion, while presenting information across specific context dependencies. Nonetheless, the mechanisms from both EICAT [10] and EICAT+ [11] can still be identified and can be associated with this classification for research or management purposes.

Each of the 19 impact types can be assigned to one of the three ecological dimensions defined in the framework (Table 1). At the ‘individual and population’ dimension, effects on individual organisms and native populations can be evaluated, such as impacts on fitness or changes in population size, which were previously addressed in EICAT. At the “species and assemblage” dimension, it is possible to assess the impact of species loss, changes in the species range, assemblage structure, successional patterns, and the soundscape. At the “ecosystem function and abiotic” dimension, broader changes to ecosystem processes and the abiotic environment can be assessed, including effects on primary production, food webs, water quality, and nutrient cycles. For each impact detected, the classification criteria are applied to determine the impact magnitude category (Table 2).

**Table 2:** EEICAT magnitude impact category criteria for classification.

Impact magnitude category	Criteria
**Minimal concern (MC)**	Impacts of minimal concern are assigned when the invasive species is present in the environment and may interact with the native biota or ecosystem, but no measurable impacts are detected at any ecological level. This includes cases where no changes in native species populations, community attributes, or ecosystem processes can be observed. General criterion: No statistically significant impact/change detected.
**Minor impact (MN)**	A minor impact is assigned when significant effects are observed at the individual level, such as changes in health, behavior, or physiology, but these do not lead to measurable changes in native population sizes. At the species and assemblage dimension, changes may include range shifts of species without any species loss or gain. At the ecosystem and abiotic dimension , abiotic alterations may be detected (e.g., changes in water chemistry or soil properties) that do not result in detectable impacts on native biota or ecological functioning. General criterion: Impacts are limited to one level and do not propagate to broader ecological levels.
**Moderate impact (MO)**	A moderate impact is assigned when effects on individuals result in changes in native population size or in genetic diversity, and these impacts are likely to be irreversible at the individual level (e.g., permanent alteration in reproductive capacity). At the species and assemblage dimension, changes in species distributions may significantly alter community metrics such as diversity or evenness. At the ecosystem and abiotic dimension, alterations in abiotic components lead to detectable impacts on native biota, and changes in ecosystem functioning are observable but remain limited in magnitude. General criterion: Impact extends to the broader ecological level with moderate severity.
**Major impact (MR)**	Major impacts involve substantial changes in native population sizes (e.g., persistent modifications or altered population dynamics) and changes to assemblage structure, including species loss or gain. These impacts are considered reversible if the invasive species is removed. At the ecosystem and abiotic dimension, major effects on ecological functions or abiotic conditions are observed but are not permanent. General criterion: Large but reversible impacts across multiple ecological levels.
**Massive impact (MV)**	A massive impact is assigned when the effects of the invasive species are permanent and not reported as reversible. This may include irreversible population genetic changes, permanent species loss or gain leading to a new community structure, complete reorganization of ecosystem functions, or major, lasting alterations to abiotic conditions (e.g., nutrient cycling, hydrology, or microclimate), even if effects on native biota are not always explicitly documented. General criterion: Irreversible impacts across one or more ecological levels.

## New features and dimensions in EEICAT

The framework introduces several new features through revised concepts and classification rules, including: an impact-based, rather than invasive species-based, approach; explicit consideration of both increases and decreases in ecological variables; the inclusion of impact levels for abiotic components and ecosystem functions; and adjusted criteria to support more consistent classification across impact levels. The aim of this final feature is to improve the reproducibility of assessments: validation work on this point is ongoing.

**Fig 1:**
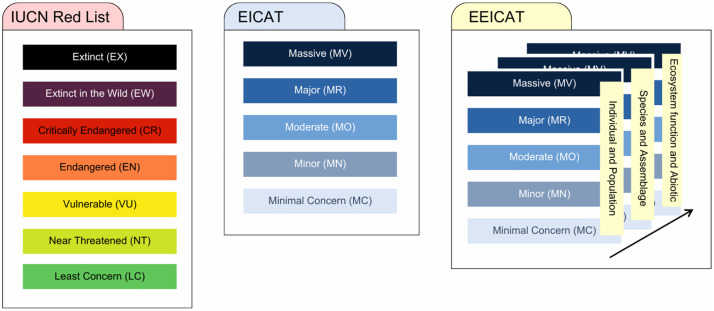
Comparison of the IUCN Red List, EICAT, and EEICAT. A comparison of the magnitude categories and dimensions used in the IUCN Red List, EICAT, and EEICAT frameworks. IUCN, International Union for Conservation of Nature; EICAT, Environmental Impact Classification for Alien Taxa; EEICAT, Extended EICAT.

EEICAT builds on the original EICAT framework [10,16,17], deliberately retaining most of the impact magnitude categories, classification criteria, and core concepts to ensure consistency. However, a fundamental distinction lies in what is being assessed. While EICAT evaluates impacts at the invasive species level, EEICAT focuses on individual invasion events, with the specific combinations of invasive species and affected systems reported in each study. Indeed, the same invasive species can lead to contrasting impacts in different invasions, which need to be accounted for. With EEICAT, assessments are based directly on measurable changes documented in the data. We call this approach impact-based assessment. In EICAT, official assessments are made at the invasive species level, regardless of individual invasion events, retaining the highest category identified from the review of all invasion events available. We call this EICAT approach a species-based assessment. Thus, EICAT attributes a single impact magnitude category to an invasive species, while EEICAT provides one or several impact magnitude categories for each impact type exerted by an invasive species in each invasion (i.e., impact event, Box 1). This not only accounts for cases where the same species can have different impacts in different invasions, but also for simultaneous or successive impacts that can be different, in space or in time. This distinction is important to avoid confusion in their applications, since the EICAT is not only endorsed by the International Union for Conservation of Nature (IUCN) but is also based on its criteria principle (Fig 1). EICAT is largely applied with this species-focus approach, including the integration of assessments into the IUCN global invasive species database, where information for each species can be retrieved at a global scale from these assessments (Fig 1). Nonetheless, our new framework can be applied in multiple ways, including: as a complimentary assessment for impacts not captured in the species already evaluated by EICAT; for a single study that investigated impacts of invasive species on native biota or the environment; for multiple studies focusing on different impacts (e.g., applications in large databases); and for recognizing that the same invasive species may have different impacts in different ecosystems, and therefore be assigned to different levels of concern and priority.

The EEICAT framework is designed to be applicable to any amount of information available in each study. This means that even a single study documenting one type of impact can be evaluated, and while more extensive datasets or complex large-scale studies can provide more information, all of them combined for each specific objective will ultimately provide broader and more detailed assessments. Since research resources are usually limited, which can prevent assessments on long-term and ecosystem-wide scales, our impact-based approach is targeted to the impact event (Box 1), which can be more useful from the management and practitioner’s viewpoint. Furthermore, it remains possible to attribute the highest recorded magnitude category from multiple EEICAT assessments to obtain a single magnitude category per invasive species and use it for EICAT. In addition, it is possible to catalogue the adequacy of evidence obtained from the vast and rapidly growing ecological impacts literature [29], including on the availability of the data and methods as well as the nature of the approach used (e.g., observation versus prediction [30]) in the assessed studies. Reporting such indicators of reliability will ensure that the breadth of evidence that can be included is maximized, while allowing users to filter impact studies based on rigor or empirical relevance. It will also allow for a more granular understanding of knowledge needs that inform future research.

Other assessments, such as SEICAT and EICAT+ [11,31], have distinct focuses: SEICAT addresses socio-economic impacts and is therefore outside the scope of our framework extension, whereas EICAT+ focuses on positive environmental impacts. In many invasion events, ecological changes are multidirectional—some species or processes increase, while others decline—therefore our extension encompasses aspects of EICAT+ by considering any directional change as an impact (Table 2). EEICAT explicitly accommodates this complexity by assigning impact categories separately to each measurable change, rather than requiring a single aggregate outcome. For example, the spread of the invasive smooth cordgrass in Chinese estuaries reduces the abundance of benthic invertebrates characteristic of mudflats while simultaneously increasing species that thrive in saltmarsh conditions [32]. Similarly, invasive bivalves, such as zebra mussels (*Dreissena polymorpha*), can reduce native unionid richness while increasing water clarity and macrophyte growth [33]. In both cases, EEICAT treats decreases and increases as distinct impacts, each classified within the relevant ecological dimension. We do not label these changes as negative or positive, as such value-laden distinctions [34] often fall outside the scope of this ecological framework. Assigning a normative value to these changes, whether desirable or undesirable, is the task of stakeholders, managers, and the community members concerned, who will need to evaluate each case within its local context.

**Fig 2:**
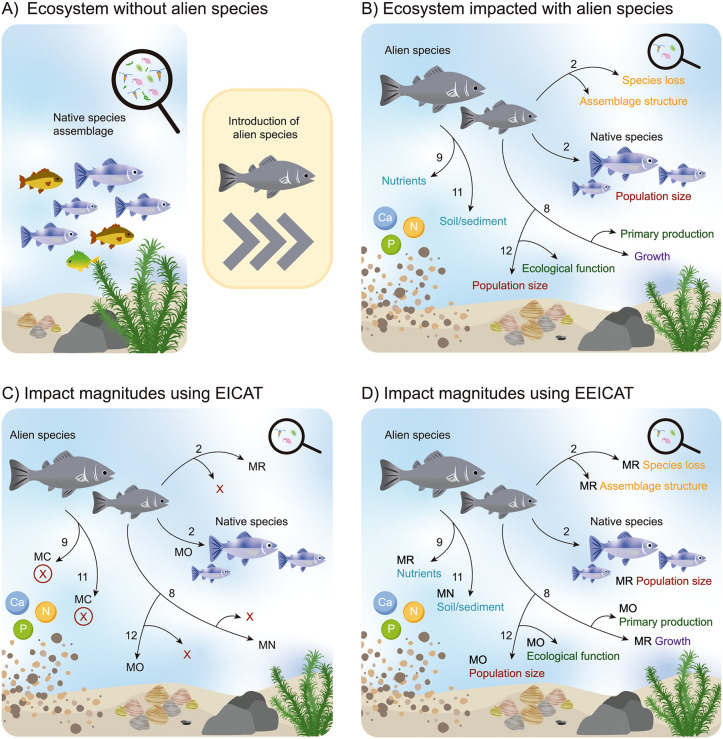
An example of EEICAT application and differences with EICAT. (**A)** An undisturbed ecosystem with native species only. (**B)** After the introduction of the invasive species (in gray), it directly interacts with native biota by predating (2) fish (i.e., declining population size), invertebrates (i.e., species loss and change in the assemblage structure), and plants (i.e., reduction/increase in primary production, reduction/increase in growth) through herbivory (8). It also directly changes abiotic components by bioturbation (11), impacting the lake’s sediment (i.e., impact on soil/sediment). The invasive fish also changes the nutrient pool and cycles (9) by its excretions and also due to bioturbation. An indirect impact (12) can be seen in the mussels, where the invasive fish, by disturbing the nutrient cycle and water quality, facilitates the increase of the mussel population, also impacting the ecological function of filtration. (**C)** Under EICAT, each interaction of the invasive species with the native biota and environment gives an impact magnitude category through a mechanism. However, several impacts cannot be categorized under the current EICAT framework. (**D)** All types of impact can be categorized under the EEICAT framework extension. **Key**. MN, MC, MR, and MO represent the impact categories, the numbers represent the EICAT impact mechanisms, and the arrows represent the interactions. A red “x” denotes an impact that cannot be classified under the EICAT protocol and a circled “x” denotes an impact that has a lower category under EICAT than it would be according to EEICAT. EICAT, Environmental Impact Classification for Alien Taxa; EEICAT, Extended EICAT; MC, minimal concern; MN, minor impact; MO, moderate impact; MR, major impact.

To reflect the broad scope of the framework, we expanded the current concept of environmental impact to include changes that do not necessarily affect native biota directly, allowing the framework to account for ecosystem functions and abiotic components that helps to capture indirect effects that have often been overlooked. It is worth noting that some ecosystem functions and abiotic impacts were already identifiable under the original EICAT framework [28]. The effects on native biota through chemical, physical, and structural (abiotic) factors are identified as mechanisms in EICAT [10], but their alteration (e.g., change in water pH) was systematically confined to the MC level, regardless of the magnitude of these effects. This shortcoming means that important information is lost regarding abiotic resource modifications from biological invasions, which are numerous, frequent, and disrupt basal ecosystem components with cascading consequences [19,27,35]. As previously demonstrated [13,15], these alterations can have profound direct and indirect impacts. The advantage of EEICAT in these cases is that it allows for the identification and attribution of the magnitude of these abiotic impacts, exactly like the others (Fig 2).

The criteria of some impact magnitude categories were adapted in EEICAT to reflect its application across the three ecological dimensions (Fig 1). In EICAT, which focuses on alien species and the progression of individual-level effects to population-level impacts, the category of impact was typically assessed based on the interaction mechanism and the number of organisms affected. In EEICAT, impact assessments rely on findings from individual studies, which can often describe effects in qualitative or quantitative terms; for example, the statistical significance of results can indicate that an impact is detected, but not necessarily how large it is. This can make it difficult to consistently judge the strength or scale of an impact if only numerical results are presented without a broader contextualization. To address this, we include optional numerical thresholds to help interpret the magnitude of change when the original study does not clearly state it. These thresholds are guided by the IUCN Red List guidelines to consider whether a decline in populations is large or not. Thus, in EEICAT, “not detectable” means no statistically significant result, while a change greater than 50% is considered ‘large’.

The IUCN Red List guidelines [36] were also used in EICAT [10] as a base to assess the reversibility concept, on which we also based ours (Fig 1). The reversibility concept in EEICAT refers to the potential for a native species (including individuals, populations, and assemblages), ecosystem function, or abiotic environment to recover after the removal of the invasive species. “Naturally reversible changes” [10] occur when the native species, ecosystem processes (e.g., nutrient cycling, primary productivity), or abiotic conditions (e.g., soil pH, water quality) are transient and likely to return to their original state within 10 years or 3 generations (whichever is longer) through natural processes (e.g., species migration, natural regeneration) or human-assisted actions that were already occurring at similar rates before the invasive species’ impact. For example, if nutrient cycling was disrupted by the invasive species, reversibility would mean the system can restore its original nutrient dynamics without additional human intervention. Reintroductions or restoration efforts requiring extra effort (e.g., active habitat rehabilitation, artificial nutrient addition) are not natural and therefore excluded. “Naturally irreversible changes” occur when the native species, ecosystem functions, or abiotic conditions cannot return within the same timeframe without significant additional human intervention [10] or even after human intervention, meaning they are permanent (e.g., the system has reached a different, stable equilibrium). This may happen due to impossible spontaneous recolonization, global extinction, or persistent environmental alterations caused by the invasive species (e.g., soil modification, altered hydrology, or irreversible changes in nutrient cycling) that prevent recovery. For instance, if an invasive species permanently alters soil chemistry or nutrient availability, the ecosystem may lose its ability to support the original community or function, making recovery naturally (or even artificially) impossible.

Because ecological impacts vary widely and data on recovery times after invasive species removal are often lacking, we include impact transience as an additional factor to help assess reversibility. This is particularly useful for abiotic components since they might be difficult to generalize under the criteria of three generations or 10 years (as thresholds applied in IUCN Red List [37]). Considerations of the reversibility of impacts under our framework should be drawn directly from the conclusion of the assessed study.

## Emerging applications of the EEICAT

Given the recognized complexities of assessing the ecological impacts of invasive species, the extension of the EICAT framework into EEICAT aims to offer a more comprehensive tool for identifying and categorizing all types of impacts across all ecological dimensions. This extended framework aims to be especially valuable for evaluating the effects of ecosystem engineer species, examining cross-ecosystem impacts, and capturing indirect effects that are often overlooked in traditional assessments.

## Ecosystem engineers

Ecosystem engineers are species that modify, create, or maintain habitats, significantly altering the availability of resources for other organisms [38]. These modifications include changes to physical structures, nutrient cycling, or hydrology, which can transform habitats and shift ecological conditions toward a new equilibrium, potentially leading to irreversible changes. Invasive ecosystem engineers are particularly concerning, as their impacts can go beyond the habitat level and trigger cascading effects on biodiversity and ecosystem function (e.g., bivalves, [13,39]).

The EEICAT framework enables researchers to evaluate how these species influence key ecological functions by explicitly accounting for changes to ecosystem processes, such as nutrient dynamics or hydrological regimes. For example, invasive bivalves that alter water turbidity, sediment composition, and nutrient cycling could be assessed not only for their biotic impacts on native species but also for their abiotic contributions to ecosystem transformation [40]. Assessments could similarly be made for many highly invasive ecosystem engineering species. For example, sour fig (*Carpobrotus edulis*) can alter coastal dunes in California, USA, by dominating the native communities and changing water, nutrient availability, and soil structure [41,42]. Similar effects are observed in many invasive ant species worldwide [43], which like plants are ecosystem engineers.

## Indirect effects

When an invasive species reduces the population size of a native species, the resulting impact is often straightforward to observe and measure. However, since species and ecosystems are interconnected, these changes can propagate through ecological networks, sometimes resulting in the demographic increase of other species (Fig 2B). While such increases might superficially appear beneficial [11,44], particularly if assessed in isolation, within an ecosystem-level framework, these effects are more accurately interpreted as disturbances.

Indirect effects can be identified and classified using the EEICAT framework, which allows for the separation of the mechanisms of impact and attribution of impact categories (Fig 2D). For example, when an invasive plant species alters nutrient availability, this may lead to changes in the microbial community, such as a shift in fungal species composition [45]. Under EEICAT, this would be represented as a direct impact on nutrient content and an indirect impact on the assemblage structure, with both impacts receiving separate impact categories.

Another example involves the invasive red swamp crayfish (*Procambarus clarkii*), which consumes aquatic plants, leading to reduced habitat complexity for other aquatic organisms and changes in water quality [46]. This direct consumption of plants impacts the physical structure of the habitat while indirectly affecting fish and invertebrate communities that rely on the vegetation for habitat and diet. Using EEICAT, both the direct impact on habitat structure and water quality, and the indirect impacts on associated species can be evaluated and assigned relevant impact categories.

## Cross-ecosystem effects

Invasive species can significantly impact ecosystems beyond their immediate habitats by altering the flow of matter and organisms across ecosystem boundaries [27]. These cross-ecosystem effects can disrupt ecological balances and affect biodiversity on a broader scale. The introduction of rats (*Rattus* spp.) to islands in the Chagos archipelago has led to a decline in seabird populations due to predation [25,26]. This reduction in seabird numbers decreases the deposition of nutrient-rich guano, which in turn affects the nutrient dynamics of adjacent coral reef ecosystems. The nutrient loss contributes to reduced fish biomass and altered fish behaviors and, ultimately, reef community structures. Using the EEICAT extension, it is possible to account for both the seabird population size alterations due to predation and the cascading effects on nutrient inputs and reef ecosystem functioning.

Similarly, plants that invade riparian zones, such as red river gum (*Eucalyptus camaldulensis*), significantly alter aquatic ecosystem processes in Botswana’s Lotsane river [47]. This invasive species shed more leachate compared to the native leadwood (*Combretum imberbe*), altering nutrient inputs. Additionally, microbial and detritivore breakdown rates are lower in the invasive leaf litter, disrupting microbial activity and the detrital decomposition process (change in ecological function). In this case, not only can changes in nutrients be attributed impact categories, but also impacts on ecological functioning can now be categorized, providing a more comprehensive view of these interconnected effects.

## Framework usage and considerations

As for EICAT assessments, applying the EEICAT framework requires at least a basic understanding of biological invasions and familiarity with the 19 types of impacts [8]. Since the protocol is impact-based, it does not require extensive research on the assessed species (S1 Fig). EEICAT can be applied to any invasion event (i.e., specific combination of invasive species, recipient system, and context), including when already documented in published studies or other reliable sources of evidence (S1 Fig). As it can be readily applied to the thousands of published studies in ecological impacts of invasions worldwide, this substantially increases the speed and scope of individual invasion assessments that can be made, broadening the range of evidence that can be integrated into the framework and made available for managers. EEICAT can also be applied progressively: even partial evidence can be recorded and categorized, providing value for synthesis and highlighting gaps.

The format wherein the assessments of impacts are reported depends on the context of application. If conducting primary research and assessing identified impacts, we recommend reporting the impact types, their ecological dimension, and their specific impact magnitude category. For details on data requirements and ideal reporting format, please refer to the supporting information (S1 Fig and S1 and S2 Tables). Our goal is to facilitate clear and independent communication of the impacts identified in each study, while accounting for variation in evidence quality, allowing readers to understand individual impact severity without needing to consult additional sources.

**Fig 3:**
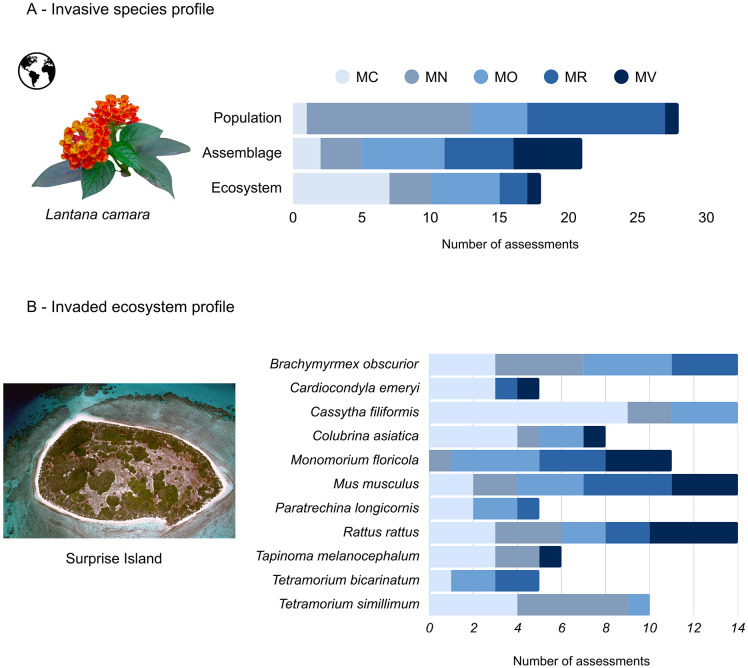
Visualizing impacts identified by EEICAT. A fictitious example of the representation of an invasive species profile (A) and an invaded ecosystem profile **(B)**, where the data comes from multiple studies assessed through EEICAT. The amount of evidence assessed for each impact category can be separated at each ecological dimension. Image credit: **(A)** Image adapted from Emerymakini (CC-0,1.0). **(B)** Aerial photograph of Surprise Island, New Caledonia, by J.-B. Duaux, used with permission. EEICAT, Extended Environmental Impact Classification for Alien Taxa.

Once data is available from multiple impacts, we propose two complementary visualization tools. The “invasive species profile” (Fig 3A) aggregates all recorded impacts caused by a single invasive species, whether from one invasion case or across multiple independent invasions. Impacts can be grouped either by ecological dimension (see the illustrative example in Fig 3) or by impact type. This format can facilitate clear communication of impact severity to managers and stakeholders, while also highlighting how impacts vary by context. We also propose the concept of an “invaded ecosystem profile” visualization (Fig 3B), which compiles impacts from different invasive species recorded within a single location or ecosystem. This is particularly useful for synthetic analyses (e.g., meta-analyses), evidence syntheses, and manager assessments. This approach also helps visualize how multiple invaders may jointly affect an ecosystem across different impact types. Both profiles enable a flexible, multidimensional view of impact data, whether across species, invasion cases, or impacted sites. The resulting profiles (Fig 3) can help stakeholders to prioritize species or ecosystems where multiple impact pathways converge, while explicitly accounting for reproducibility.

As a concrete example, we analyzed a representative subset of studies of invaded protected areas with high confidence (i.e., both methods and data available), to illustrate how several separate sources of impact evidence can be grouped to be analyzed in a broader synthesis [19] (Table 3). A key improvement in EEICAT is the ability to assign impact magnitude categories to changes in ecosystem functions, which EICAT did not cover [40]. For example, the invasive nitrogen-fixing tree Moluccan albizia (*Falcataria moluccana*) alters decomposition rates and nitrogen cycling, potentially leading to long-term shifts in ecosystem configuration. These changes are critical for management, particularly in nutrient-poor environments where native species are adapted to specific conditions. Similar functional impacts are seen in molasses grass (*Melinis minutiflora*) through altered fire regimes, and in species like wild boar (*Sus scrofa*) and common carp (*Cyprinus carpio*), which affect soil structure and water quality, respectively. Recognizing and categorizing these impacts supports more informed decisions in conservation planning and ecosystem management in protected areas.

**Table 3:** Examples of EEICAT application on studies assessing impacts of invasive species in protected areas.

Study	Protected area	Country	Invasive species	Impacted recipient	Variable measured	Impact type	EEICAT category
Hughes and colleagues [48]	Keauohana Forest Reserve	USA	*Falcataria moluccana*	*Metrosideros polymorpha*	Decomposition	Ecological function	MR
					Soil nitrogen	Nutrient pool	MO
Rossi and colleagues [49]	Serra do Rola-Moça State Park	Brazil	*Melinis minutiflora*	Plant community	Biomass	Primary production	MR
					Richness	Assemblage structure	MR
					Fine fuel loads	Fire regime	MN
Xu and colleagues [50]	Tianmushan National Nature Reserve	China	*Phyllostachys edulis*	Soil microbiota	Diversity	Assemblage structure	MO
				Plant community	Soil pH	Soil sediment	MN
					Nutrient content	Nutrient pool	MN
Krull and colleagues [51]	Waitakere Protected Private Land	New Zealand	*Sus scrofa*	Nutrient	Soil nitrogen	Nutrient pool	MO
				Litter cover	Litter cover	Soil sediment	MC
				Plant community	Seedling survival	Health/growth	MC
					Richness	Assemblage structure	MO
Angeler and colleagues [52]	Tablas de Daimiel National Park	Spain	*Cyprinus carpio*	Water parameter	Dissolved oxygen	Water quality	MC
				Copepods	Density	Assemblage structure	MO
				Zooplankton	Biomass	Population size	MO
				Zooplankton	Food web	Food web	MO
				Phytoplankton	Chlorophyll-a	Primary production	MR

MC, minimal concern; MN, minor impact; MO, moderate impact; MR, major impact.

Importantly, EEICAT does not prescribe management actions directly; instead, it provides an evidence-based foundation that, if needed, can be combined with socio-economic or value-based frameworks (such as SEICAT) to guide decision-making. Moreover, as an impact-based approach, EEICAT improves transparency, reduces subjective interpretation, and allows specific context-dependence assessments. Thus, managers could select the invaded ecosystem profile, the species ecosystem profile, or the invasion event assessments according to their specific problems at the local scale. Compared with the status quo, the novelty of EEICAT with respect to previous frameworks relies precisely on the availability of impact-based assessments together with the characteristics of the invasion event, which should help to improve management decisions, especially prioritization [19].

## Conclusions

As biological invasions and their impacts continue to expand globally, there is an urgent need for tools that can assess not only species-level effects but also the broader ecological consequences across directionality, space, function, and time. While the original EICAT framework laid the foundation for categorizing species-level impacts, it did not fully capture the cascading and systemic effects that invasions can trigger. With the proposition of EEICAT, we expand the EICAT framework by including the many missing impact types at the assemblage, ecosystem, and abiotic levels. By doing so, we apply a complex network logic that recognizes that species in ecosystems are functionally interdependent and therefore, that species-level alterations inevitably amplify throughout the system. Relying on the same logic, the EEICAT framework focuses on invasion events rather than invasive species, allowing assessment of multiple impacts and their aggregation within and across invasive species, ecological scales, and/or invaded habitats and regions, as well as considering their temporal dynamics. Our approach aims to help identify research gaps and support the prioritization of management efforts, particularly for invasive ecosystem engineers or those impacting key ecosystem processes. While we do not aim to provide an exhaustive list of possibilities here, we have highlighted how EEICAT can advance our understanding of the ecological impacts of biological invasions.

## Supporting information

S1 Fig Assessment process for EEICAT. The four-step process of assessment using EEICAT. (1) Identification of the studies reporting ecological impacts of invasive species; (2) identification of the impacts caused by each invasive species for each impacted recipient; (3) evaluation of the evidence reported, particularly the reproducibility, results and other evidence of the impacts identified (at this stage, assessors can also identify and report mechanisms following EICAT and EICAT+ guidelines); (4) classification of the invasion event, i.e., each impact — invasive species — recipient combination, in one of the five impact magnitudes in its appropriate dimension (i.e., population, assemblage or ecosystem).
(TIFF)

S1 Table Example of impacts classification of *Alliaria petiolate.* (DOCX)

S2 Table Recommended minimum set of information reporting for EEICAT. (DOCX)

## Abbreviations

EICAT: Environmental Impact Classification for Alien Taxa
EEICAT: Extended Environmental Impact Classification for Alien Taxa
IUCN: International Union for Conservation of Nature
MC: minimal concern
MN: minor impact
MO: moderate impact
MR: major impact
MV: massive impact
