# Supplementary figures and images for: Expanding invasive species impact assessments to the ecosystem level with EEICAT
Source: PLoS Biol. 2026 Mar 10;24(3):e3003665. doi: 10.1371/journal.pbio.3003665 (PMC12974798; doi:10.1371/journal.pbio.3003665)

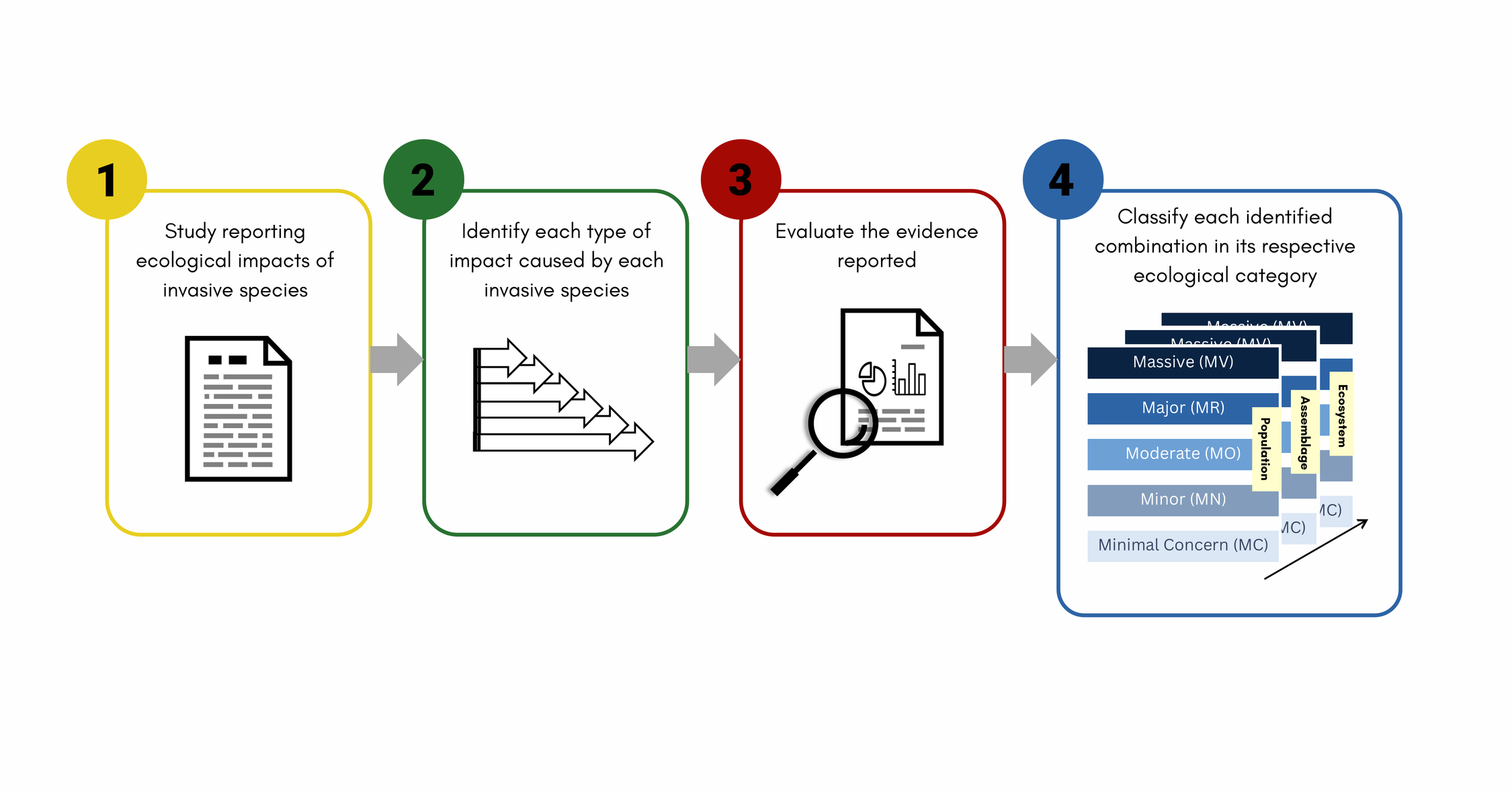

Supplement: S1 Fig — The four-step process of assessment using EEICAT. (1) Identification of the studies reporting ecological impacts of invasive species; (2) identification of the impacts caused by each invasive species for each impacted recipient; (3) evaluation of the evidence reported, particularly the reproducibility, results and other evidence of the impacts identified (at this stage, assessors can also identify and report mechanisms following EICAT and EICAT+ guidelines); (4) classification of the invasion event, i.e., each impact — invasive species — recipient combination, in one of the five impact magnitudes in its appropriate dimension (i.e., population, assemblage or ecosystem). (TIFF) [file pbio.3003665.s001.tiff]
