# Supplementary material for: Expanding invasive species impact assessments to the ecosystem level with EEICAT
Source: PLoS Biol. 2026 Mar 10;24(3):e3003665. doi: 10.1371/journal.pbio.3003665 (PMC12974798; doi:10.1371/journal.pbio.3003665)
Supplement: S1 Table — (DOCX) [file pbio.3003665.s002.docx]

**Supporting information**

**Expanding invasive species impact assessments to the ecosystem level with EEICAT**

**S1 Table. Example of impacts classification of *Alliaria petiolata.***

| **Reference** | **Invasive species name** | **Impacted recipient name** | **Variable measured** | **Impact type** | **EEICAT** |
| --- | --- | --- | --- | --- | --- |
| Callaway et al., 2008 | *Alliaria petiolata* | Soil | Release of allelopathic components | Soil sediment | MR |
|  |  | Fungal community | Diversity | Assemblage strucuture | MV |

Note: The table and examples provided are illustrative and not exhaustive; users should adapt them to the specific context of their study. EEICAT allows reporting of mechanisms following EICAT and EICAT+ guidelines and can accommodate temporal aspects when impacts are measured over time. EEICAT impact codes (e.g., MR = Minor, MV = Moderate) are used throughout and should be interpreted according to the main framework definitions.

Example of EEICAT application step by step:

- *Step 1: Study reporting ecological impacts of invasive species:* Garlic mustard (*Alliaria petiolata*), is an invasive plant that releases antifungal chemicals, eliminating native arbuscular mycorrhizal fungi, and causing suppression of the native tree seedling growth [1–3] This disruption affects both biotic interactions and abiotic processes, illustrating assemblage and ecosystem-level impacts.
- *Step 2 and 3: Identification of ecological impact types and the evidence reported:* By applying the impact type classification [4], the impacts from the garlic mustard can be identified on soil or sediment modification (from the release of allelopathic components in soil), change in the assemblage structure (from reduced diversity in fungal community) and impacts on health and/or growth (tree seedling suppression). At this stage, it is recommended to assess reproducibility in the evidence provided. Specific guidance can be found in Tables S2.
- *Step 4: Classification of each combination invasive species x impact type:* the table structure (S1 Table) that can be used to report the impacts and their classification. Please note that this is not a complete assessment for this study.

When applying EEICAT framework it is necessary to report the following set of information (and to consult the EICAT guidelines [5] for a full set of other recommended descriptors):

Table S2: Recommended full set of information reporting for EEICAT

|  | **Description** |
| --- | --- |
| **Source of information** | Full reference and document title. |
| **Type of information** | for example, studies with experimental, quasi-experimental, observational, or descriptive, reports from local communities. |
| **Time frame** | period during which the study was conducted. |
| **Invasive species** | taxon assessed (with scientific and common name). |
| **Impacted recipient** | the native species, community, or ecosystem component affected. |
| **Region and location** | country, ecosystem, and geographical coordinates if available. |
| **Impact type** | the type of ecological impact detected, following Carneiro et al., 2025 |
| **Impact severity** | the magnitude of the effect as assessed within EEICAT. |

**References**

1. Callaway RM, Cipollini D, Barto K, Thelen GC, Hallett SG, Prati D, et al. Novel Weapons: Invasive Plant Suppresses Fungal Mutualists in America but Not in Its Native Europe. Ecology. 2008;89: 1043–1055. doi:10.1890/07-0370.1

2. Stinson KA, Campbell SA, Powell JR, Wolfe BE, Callaway RM, Thelen GC, et al. Invasive Plant Suppresses the Growth of Native Tree Seedlings by Disrupting Belowground Mutualisms. PLOS Biology. 2006;4: e140. doi:10.1371/journal.pbio.0040140

3. Volery L, Blackburn TM, Bertolino S, Evans T, Genovesi P, Kumschick S, et al. Improving the Environmental Impact Classification for Alien Taxa (EICAT): a summary of revisions to the framework and guidelines. NeoBiota. 2020;62: 547.

4. Carneiro L, Leroy B, Capinha C, Bradshaw CJA, Bertolino S, Catford JA, et al. Typology of the ecological impacts of biological invasions. Trends in Ecology & Evolution. 2025;0. doi:10.1016/j.tree.2025.03.010

5. Guidelines for using the IUCN Environmental Impact Classification for Alien Taxa (EICAT) Categories and Criteria. Gland, Switzerland and Cambridge: IUCN; 2020.
