# Supplementary material for: Expanding invasive species impact assessments to the ecosystem level with EEICAT
Source: PLoS Biol. 2026 Mar 10;24(3):e3003665. doi: 10.1371/journal.pbio.3003665 (PMC12974798; doi:10.1371/journal.pbio.3003665)
Supplement: S2 Table — (DOCX) [file pbio.3003665.s003.docx]

**Supporting information**

**Expanding invasive species impact assessments to the ecosystem level with EEICAT**

When applying EEICAT framework it is necessary to report the following set of information (and to consult the EICAT guidelines [1] for a full set of other recommended descriptors).

**S2 Table. Recommended minimum set of information reporting for EEICAT**

|  | **Description** |
| --- | --- |
| **Source of information** | Full reference and document title. |
| **Type of information** | For example, studies with experimental, quasi-experimental, observational, or descriptive, reports from local communities. |
| **Supporting data** | Presence of data supporting the impact event. |
| **Description of methods** | Presence of methods used to test the impact. |
| **Time frame** | Period during which the study was conducted. |
| **Invasive species** | Taxon assessed (with scientific and common name). |
| **Impacted recipient** | The native species, community, or ecosystem component affected. |
| **Region and location** | Country, ecosystem, and geographical coordinates if available. |
| **Impact type** | The type of ecological impact detected, according to the standardized typology [2]. |
| **Impact mechanism** | The mechanism through which an invasive species exerts its impact, according to [1,3]. |
| **Impact severity** | The magnitude of the effect as assessed within EEICAT. |

**References**

1. Guidelines for using the IUCN Environmental Impact Classification for Alien Taxa (EICAT) Categories and Criteria. Gland, Switzerland and Cambridge: IUCN; 2020.

2. Carneiro L, Leroy B, Capinha C, Bradshaw CJA, Bertolino S, Catford JA, et al. Typology of the ecological impacts of biological invasions. Trends in Ecology & Evolution. 2025;0. doi:10.1016/j.tree.2025.03.010

3. Vimercati G, Probert AF, Volery L, Bernardo-Madrid R, Bertolino S, Céspedes V, et al. The EICAT+ framework enables classification of positive impacts of alien taxa on native biodiversity. PLOS Biology. 2022;20: e3001729. doi:10.1371/journal.pbio.3001729
